# Effect of Polyethylene Glycol and Humic Acid Coating on NPK Release From Controlled-Release Fertilizer

**DOI:** 10.1155/2024/5510660

**Published:** 2024-11-21

**Authors:** Suwardi, Dyah Tjahyandari Suryaningtyas, Abdul Ghofar, Mochamad Rosjidi, Anwar Mustafa, Hens Saputra

**Affiliations:** ^1^Division of Physical Land Resource Development, Department of Soil Science and Land Resources, Faculty of Agriculture, IPB University, Bogor, Indonesia; ^2^Center for Mine Reclamation Studies, International Research Institute for Environment and Climate Change, IPB University, Bogor, Indonesia; ^3^Research Centre for Process and Manufacturing Industry Technology, National Research and Innovation Agency, Bogor, Indonesia

**Keywords:** ammonium, controlled-release fertilizer, humic acid, nitrate, polyethylene glycol

## Abstract

Plants require essential macronutrients such as nitrogen (N), phosphorus (P), and potassium (K), but their availability in soil is often inefficient due to evaporation, leaching, and binding. Controlled-release fertilizers (CRFs) provide a solution by regulating nutrient release over time. This study evaluates the effects of two coating materials, polyethylene glycol (PEG) and humic acid, on the release patterns of N, P, and K during an 18-week incubation using Inceptisol from Bogor, Indonesia. Various CRF treatments were tested, including uncoated (A1, A2), PEG-coated (B1, B2), and humic acid–coated (C1, C2) formulations. Results showed that CRF with PEG (B2) demonstrated slower N release, with ammonium levels decreasing from 32.22% in week 1 to 9.36% by week 18. Nitrate release increased steadily from 26.37% to 37.36% between weeks 3 and 18. In contrast, CRF with humic acid (C2) showed slower nitrate release, reaching 36.26% by the end of incubation. P release patterns were similar across treatments, while K release was lowest in the humic acid–coated treatment (C2) at 24.48%. These findings underline the potential of coating materials like PEG and humic acid to optimize nutrient release, enhancing agricultural efficiency.

## 1. Introduction

Nitrogen (N), phosphorus (P), and potassium (K) are essential nutrients needed by plants in large amounts. However, the availability of these elements in the soil is often found in very small amounts [1, 2]. N is an important nutrient because it contains proteins, nucleic acids, and other essential elements [3]. N is taken up by plants from the soil solution in the form of NO_3_-N or NH_4_-N. N fertilizer as a source of N in the soil with the support of microorganisms will be converted through the ammonification process to NH_4_^+^ and the nitrification process from NH_4_-N to NO_3_-N [4–6]. N can accelerate vegetative growth such as formation of tillers, plant height, and leaf width and increase plant protein content [7–9].

The loss of N in the soil is not only due to evaporation, leaching, and erosion but also through the denitrification process [10]. Reference [11] stated that denitrification had the highest activity in the surface soil and contributing up to 75% of the N loss. Soil with a high clay content experiences N loss through the denitrification process that generally occurs when plants are irrigated after fertilization [12]. Irrigation is used to set the upper boundary condition, while groundwater levels define the lower boundary condition. The process accounts for root water uptake, crop N uptake, N leaching, nitrification, and denitrification [13]. This causes N to be the most numerous elements added to the soil but has a low utilization efficiency level by plants [1, 14, 15].

P is absorbed by plants in the form of H_2_PO_4_^−^ and HPO_4_^2−^ ions from the soil solution [16]. P stores and transfers energy in all plant metabolic processes, promotes seedling and root development, and accelerates flowering and ripening [17]. However, the availability of P in the soil is often limited as it strongly binds to other elements [18]. For instance, Fe-P and Al-P bind in low-pH soils, while Ca-P binds in high-pH soils [19].

K contained in the soil can facilitate in the process of transporting products, strengthening cell walls, activating enzymes in all plant metabolic processes, and delaying leaf aging with improving the tolerance to stresses [20, 21]; [22]. The availability of K in the soil is generally low, with only about 0.1–0.2% of the total soil K being present in a soluble form that plants can readily absorb and use [23]. In addition, the available form of K is easily leached by runoff and dramatically influenced by soil conditions [24, 25].

Plantation crops, especially annual crops, require large amounts of nutrients for a long period of time [26]. Fulfillment of annual plant nutrients is done gradually in stages in order to meet the needs during the planting period. The fertilization system and dose according to the needs of the plant species is one way to overcome nutrient deficiencies for plants [27, 28]. In addition to regulating the fertilization system, it is necessary to increase the efficiency of fertilization so that the nutrients in the fertilizer are not rapidly lost during the period of plant growth [29, 30].

Fertilizer is a supporting material in fulfilling nutrients, but fertilizer given to plants in general will experience losses caused by surface runoff, evaporation due to high environmental temperatures, fixation by soil, leaching, and microbial activities [31, 32]. Therefore, conventional fertilizers used by farmers are considered inefficient. One of the efforts that can be made to increase fertilization efficiency is improving fertilization application techniques and also the physical and chemical properties of fertilizers by controlling the nutrient solubility system and formulation of fertilizer nutrient levels [33]. Through this effort, it is hoped that the solubility and the release of nutrients can be more regulated so that the factor of nutrient loss can be reduced [34].

Controlled-release fertilizer (CRF) can manage the release of nutrients and increase nutrient uptake efficiency (NUE) [35]. According to [36], the benefits of CRF include improved nutrient efficiency, extended nutrient release, lower labor costs, reduced plant toxicity, and added macro- and micronutrients beyond NPK. CRF has constituent components that are able to release nutrients in longer period, one of which is the type of coating [37].

CRF can generally be coated with various materials, such as humic acid, starch, polyethylene glycol (PEG), silica, and other materials that regulate the rate of nutrient release [38, 39]. According to [40], release adjustment of N in urea can be made by coating, using a polymer to limit the rate of urea solubility. Using polymer coatings on CRF can greatly improve the efficiency of fertilizer usage and offer a practical solution to the issues of environmental contamination from low fertilizer utilization rates and significant losses [41]. This is particularly important, as studies have shown that plant responses to nutrient availability are linked to their biomass allocation and abiotic stress responses [42, 43].

PEG is a synthetic polymer of oxyethylene. This polymer is biodegradable, hydrophilic, and easily soluble in various solvents, has low melting point and toxicity, and is in a semicrystalline form. PEG is considered to be inert or exhibit low toxicity in animals and humans [44]. PEG can increase the nutrients absorbed in fertilizers, including nitrate from N fertilizers [45]. Fertilizers coated with biodegradable materials not only increase the nutrients absorbed in the fertilizer but also avoid damage to the soil physical and chemical properties as a result of nutrient loss [46].

Humic acid can be used as soil conditioner or ameliorant, due to its ability to stimulate and activate biological and physiological processes in living organisms in the soil [47]. Humic acid is a polyelectrolyte macromolecule containing various types of functional groups with different pKa values. The pKa value of a functional group is the negative logarithm of its acid dissociation constant (Ka), which quantifies the strength of an acid in solution. It represents the pH at which half of the functional groups are dissociated and half are in their protonated form. This property affects the solubility, reactivity, and interaction of humic acid with other soil components and microorganisms. Humic acid is able to increase the ability of the soil to bind, absorb and exchange cations, and form complex compounds with heavy metals and clay and can also provide nutrients such as N, P, K, and S into the soil and C as an energy source for soil microbes [48–51]. Reference [52] also stated that humic acid can interact with inorganic substances such as clay and metals and form a clay–metal humic acid complex. Humic acid also has a high C/N ratio indicating that the humic substance is relatively resistant to microbe degradation. Fertilizers coated with humic acid have a higher absorption rate and will stimulate the development of plant roots [53].

Recent studies emphasize the role of antioxidant defense mechanisms in mitigating abiotic stresses such as heavy metal toxicity and salinity [54]. Biomass allocation, along with plant growth regulation, forms a fundamental response to these stresses, and innovations in nutrient management strategies, such as CRF formulations, are crucial in enhancing crop resilience under stress conditions.

The objective of this study is to evaluate the effect of PEG and humic acid coatings on NPK granule fertilizers on the pattern of N release from urea in the form of ammonium (NH_4_^+^) and nitrate (NO_3_^−^), as well as P and K nutrients observed over a period of 18-week incubation. This research is an invention of a specific fertilizer formula for oil palm in the form of granules using a zeolite matrix.

## 2. Materials and Methods

This research was conducted for 18 weeks at the Laboratory of the Department of Soil Science and Land Resources, Faculty of Agriculture, IPB University. The soil sample used for this incubation study was Inceptisol taken from the Cikabayan Educational Field, Dramaga, Bogor. Inceptisol is a soil type with the most widespread land in Indonesia. Inceptisols, formed from volcanic tuff, are low in nutrients but are widely used to meet local needs, including agriculture and plantation cultivation [55, 56]. The land is planted with oil palm and gets its water from rainwater [57]. The soil samples were taken compositely at a depth of 0–20 cm. Soil samples taken were then air-dried, ground, and then sieved using a 2 mm sieve for chemical analysis.

**Figure 1:**
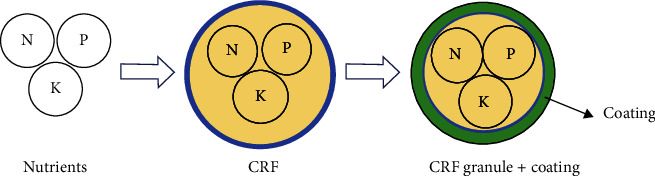
Schematic of preparing the CRF granule.

CRF granule fertilizer uses natural zeolite as a matrix and clay as a binder. Zeolite that has hollow structure may be used as a matrix of humic acid and as an ameliorant material [58]. In addition, zeolite has a high cation exchange capacity (CEC) value that can maintain a low EC value, resulting in better fertilization and absorption efficiency [59]. The optimal production of CRF granules is affected by the size of the zeolite raw material used [60]. The manufacturing process uses fuse technology at room temperature which consists of size reduction, granulation, and product drying processes. Size reduction is done using a crusher. The granulation process is carried out using a pan granulator, while drying is done using a rotary dryer. The next stage is the coating process on CRF granule fertilizer with the spray method. The scheme for making PEG or humic acid–coated CRF granulated fertilizer is shown in Figure 1.

The treatments consisted of 4 types of fertilizer, each of which consisted of 2 doses, namely, 0.31 g/100 g soil and 0.62 g/100 g soil. There were three types of CRF used in this study, containing urea, DAP, and KCl, with a composition of NPK 13:6:27. The difference between these three fertilizers is in the type of coating for each fertilizer, namely, without coating (CRF A1 and CRF A2), PEG coating (CRF B1 and CRF B2), humic acid coating (CRF C1 and CRF C2), and a comparison with NPK Mutiara 16:16:16 as presented in Table 1.

**Figure 2:**
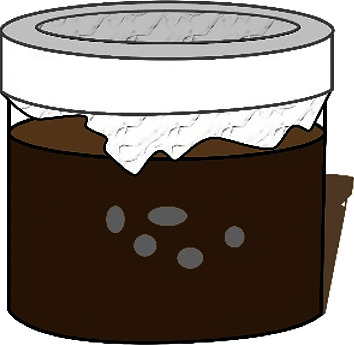
Illustration of the incubation bottle.

Measurement of fertilizer release rate was carried out using the incubation method in open space. The air-dried soil was then weighed as much as 124.47 g or the equivalent of 100 g (dry weight) with a soil moisture content of 24.47%. The soil is then put into a plastic container in a cylindrical tube with a diameter of 6.00 cm and a height of 6.70 cm. The treatments consisted of 4 types of fertilizer, each of which consisted of 2 doses, namely, 0.31 g/100 g soil and 0.62 g/100 g soil. Each treatment was added to the soil in the incubation bottle and 31 mL of water was added to reach the field capacity water content (61.66%). Soil and fertilizer in incubation bottles were covered with polyethylene plastic as shown in Figure 2.

Incubation was carried out in an open room for 18 weeks at room temperature for a certain period of time, namely, at weeks 1, 2, 3, 4, 6, 8, 10, 13, 16, and 18; during the incubation period, analysis of ammonium N (NH_4_-N), nitrate N (NO_3_-N), P, and K was carried out. The materials used in this incubation test include aquadest, NH_4_F, NaOH, HCl, KCl, PB solution, PC solution, boric acid, Conway indicator, and devarda's alloy.

Chemical analysis was conducted to determine available N, P, and K levels. The Kjeldahl method was used for N analysis, while the Bray I method was used for P and K analyses. The tools used for chemical analyses of soil samples include flame photometer, spectrophotometer, soil oven, shaker machine, analytical balance, distillation apparatus, and burette.

The data obtained are the concentration values of available-N (NH_4_-N and NO_3_-N), available-P, and available-K in ppm. The results of the measurement of the concentration value of each treatment are then reduced by the control concentration value, so that the result of the reduction is the result of the concentration of the released nutrient. The graph of the pattern of release of each nutrient is obtained from the concentration results released by each nutrient per a certain period of time which is then divided by the total concentration that has been given to the soil.

The statistical analysis for this study was conducted to compare the differences between treatments and analyze the trends over time. Initially, exploratory data analysis was performed using line graphs to visualize data distribution and initial trends. A two-way ANOVA was utilized to assess the main effects of fertilizer type (CRF A, CRF B, CRF C, and NPK Mutiara) and dosage (0.31 g/100 g soil and 0.62 g/100 g soil), along with their interaction. Significant differences identified by ANOVA were further explored using Tukey's HSD post hoc test. All analyses were performed using the statistical software Minitab.

## 3. Results and Discussion

### 3.1. N Release Pattern (NH_4_-N and NO_3_-N)

**Figure 3:**
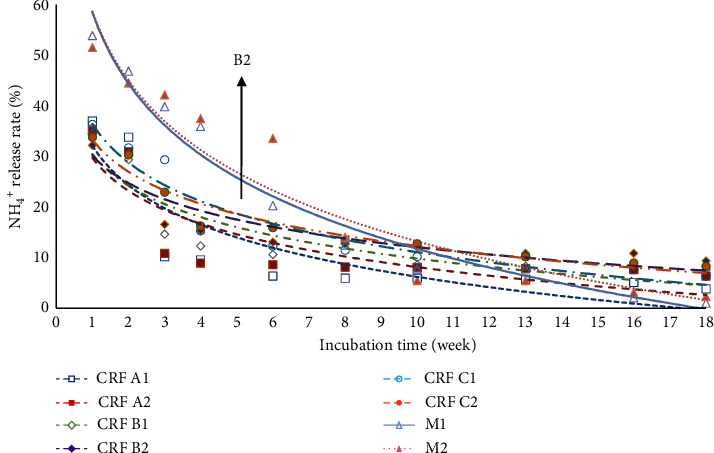
The release rate pattern of ammonium (NH_4_^+^) of the eight treatments within 18-week incubation time.

The pattern of release of ammonium (NH_4_-N) from eight fertilizer treatments during 18 weeks of incubation time is presented in Figure 3. During the first 8 weeks of fertilizer treatment, N was released in the form of ammonium, ranging from 32% to 54%. After this period, all fertilizer treatments gradually decreased, reaching levels between 1% and 9% by the 18th week of incubation.

The pattern for ammonium release in the M2 treatment had a greater gradient than the curves of the other six CRF treatments (Figure 3). Over the 18-week period, the release of N in the M2 treatment was notably faster, ranging from 51.56% to 2.34%. Statistical analysis indicated that M2 had a significantly higher ammonium release (Table 2). Previous research by Liu et al. [61] also stated that the use of slow-release N fertilizers can reduce N losses and improve N-use efficiency compared to conventional N fertilizers.

In addition to the effect of coating, it is also influenced by differences in the amount of N added to the soil. The six CRF-treated fertilizers had N content of 13% which was equivalent to 40.3 mg N at dose 1 and 80.6 mg N at dose 2. NPK Mutiara fertilizer had N content of 16% which was equivalent to 49.6 mg N at dose 1 and 97.6 mg N at dose 2. The difference in the amount of added N also affects the release of N that occurs in the soil. The application of fertilizers with higher N content also releases high amounts of N [62]; [63].

B2 treatment (CRF 13-6-27 coated with PEG at a dose of 0.62 g/100 g soil) released ammonium slightly slower than other fertilizer treatments (Figure 3). The fertilizer released 32.22% ammonium in the first week and continued to 9.36% in the 18th week of incubation (Table 2). The release of ammonium from B2 treatment was slower due to the presence of a PEG coating on the fertilizer. PEG has a hydroxyl group that is able to bind water, but PEG also has hygroscopic properties so that less urea is converted into ammonium because water is first absorbed by PEG before water is able to dissolve the urea in the fertilizer [64, 65].

However, the statistical analysis in Table 2 indicates that CRF A1 had a lower mean ammonium release over 18 weeks compared to CRF B2. Despite CRF B2 being coated with PEG, which is intended to slow ammonium release, it ultimately resulted in a higher mean release. This can be explained by PEG's hygroscopic properties, which initially slow down urea dissolution but eventually lead to a more sustained ammonium release. CRF A1 had a high initial release (37.01%) in the first week that declined rapidly, while CRF B2 had a moderate initial release (32.22%) with a higher sustained release rate. The cumulative effect of CRF B2's more evenly distributed release resulted in a higher mean over time. Therefore, despite PEG's initial slowing effect, its controlled release mechanism led to higher overall ammonium release for CRF B2, whereas CRF A1's uncoated formulation could not sustain its initial release rate.

In addition, the Tukey test shows that the difference in mean ammonium release between CRF B2 and CRF A1 (with a *p* value of 0.827) is not statistically significant (Table 3). This means that although there is a difference in ammonium release between the two treatments, it is not large enough to be considered statistically significant.

In the first week, eight of the tested fertilizers quickly turned into ammonium. The fertilizer applied to the soil will initially turn into NH_4_-N through the ammonification process which causes the ammonium ion value in the first week to show a high value. NH_4_^+^ is formed from the ammonification process which is the process of forming ammonium compounds from organic matter [66]. NH_4_^+^ produced from the ammonification process will turn into nitrate ions (NO_3_^−^) through the nitrification process. The nitrification process can only take place under conditions of sufficient oxygen (aerobic). The authors of [67] stated that the first step of the nitrification reaction involves obligate autotrophic bacteria known as *Nitrosomonas* which produces nitrite ions, and then the nitrite ions are converted into NO_3_^−^ by *Nitrobacter* bacteria. The activity of *Nitrosomonas* and *Nitrobacter* increased the amount of NO_3_^−^ in the soil formed through the nitrification process, so that the NO_3_^−^ increased in line with the incubation time.

**Figure 4:**
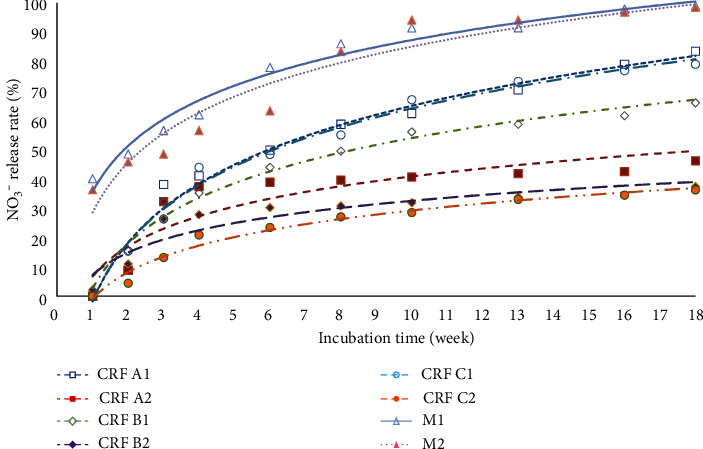
The release rate patterns of nitrate (NO_3_^−^) of the eight fertilizer treatments during 18 weeks of incubation.

The pattern of NO_3_-N release in the first week of incubation of the eight fertilizer treatments ranged from 0% to 40%, and then there was a gradual increase up to 36.26% to 98.96% at the 18th week of incubation. The CRF C2 treatment (CRF humic acid coating dose of 0.62 g/100 g soil) released NO_3_-N slightly and slowly at 36.26% until the end of the incubation period. This is supported by statistical analysis, which showed that C2 had the lowest mean release and was significantly different from other treatments (see Figure 4).

Fertilizer with treatment A1 (CRF 13-6-27 uncoated, applied at a dose of 0.31 g/100 g soil) released NO_3_-N faster than other CRF treatments; this is because CRF A1 fertilizer is CRF without coating so that the elements are more easily released into the soil. It is aligning with the statistical analysis, indicating significant differences between A1 and other CRF treatments. Previous study by Jariwala et al. [68] found that CRF can prevent direct exposure of fertilizer granules to soil and prevent loss of nutrients such as NO_3_^−^ and N_2_O emissions. Additionally, Handayani et al. [69] stated that CRF formulations with coatings are more resistant to water leaching compared to non-CRF, leading to lower nutrient solubility.

Interestingly, the release of NO_3_-N in the C1 treatment, with a humic acid coating at the same dose as A1, was similar to A1, suggesting that the C1 dose might not have been optimal for NO_3_-N release. This similarity was supported by analytical statistics, indicating no significant difference between the two treatments. This suggests that the C1 dose might not have been optimal for NO_3_-N release. However, doubling the dose in C2 (0.62 g/100 g soil) effectively optimized NO_3_-N release, as demonstrated in Table 4. Despite higher NH_4_-N production in treatments with humic acid, it did not convert to NO_3_-N in C2, possibly due to stable covalent bonds formed by NH_4_-N. The slower release observed in C2 could also be attributed to the larger fertilizer dose or the presence of functional groups on humic acid forming stable bonds. The authors of [70] stated that humic substances are characterized by a variety of interactions and chemical bonds that contribute to their stability. The bonds in humic substance are particularly difficult to break down, and their structural units are highly diversified.

The NPK Mutiara fertilizer treatments (M1 and M2) exhibited the highest release of NO_3_-N, consistent with the statistical analysis results as shown in Table 4. This can be attributed to the higher NO_3_-N content in Mutiara fertilizers (6.5%), leading to a rapid release of NO_3_-N during the initial weeks of incubation. Unlike CRF nitrate release, which involves nitrification processes, the NO_3_-N released from Mutiara fertilizers originates directly from its raw materials.

**Figure 5:**
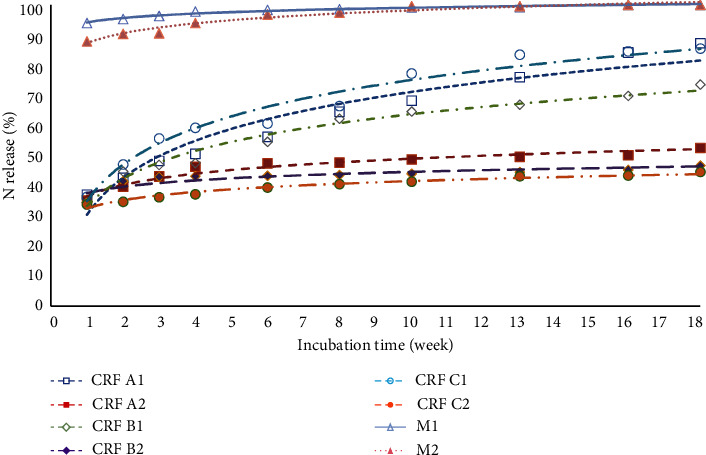
The release pattern of nitrogen (%) of the eight fertilizer treatments during 18 weeks of incubation.

The findings presented in Figure 5 demonstrate variation patterns of N release among different treatments during the incubation period. Specifically, A1, B1, and C1 display an increasing trend in N release over time; meanwhile, A2, B2, and C2 show relatively low N release rates. M1 and M2, on the other hand, demonstrate consistently high N release rates from the beginning of the incubation period, with a relatively constant release rate after that. These observations align with the statistical analysis results presented in Table 5, indicating that M1 and M2 have the highest mean values and show significant increases in N release over time (Table 5).

The amount of fertilizer applied to soil can affect the rate of N release [3]. Specifically, CRF with a humic acid coating dose of 0.31 g/100 g (C1) demonstrates a relatively rapid N release compared to other CRF variants. While C1 shows the highest mean among CRF variants, statistical analysis indicates that it does not show significant differences compared to other CRF variants at the same dosage (A1 and B1). However, it does show significant differences when compared to A2 and B2 (Table 5). In contrast, CRF with a humic acid coating at multiple dosages (C2) has a slower N release rate. This is supported by the lowest mean difference significant letter, indicating that increasing the fertilizer dose slows down N release. This suggests that at a certain dose, the N release from CRF with a humic acid coating results in better efficiency [71]. A study by Suwardi [72] on the effect of humic substances on macronutrients in the soil showed that total N increased with the treatment of humic substances.

CRFs with PEG coatings (B1 and B2) release N (N) more slowly compared to A1 and A2 due to the presence of PEG coatings, which could prevent the ammonification process within the fertilizers. This slowdown in the ammonification process subsequently delays the nitrification process, resulting in reduced N release rates compared to A1 and A2 without coatings. Furthermore, the M-treated fertilizer has higher N release pattern than the CRF-treated fertilizers. This difference is derived from the fact that M fertilizer is not a CRF, so it does not have mechanisms to regulate nutrient release. As a result, N-NH_4_^+^ and N-NO_3_^−^ are rapidly released during the incubation period. This finding is consistent with a study in [73] which stated that SRF can slow down the release of ammonium and nitrate compared to Mutiara NPK (16-16-16) fertilizer.

**Figure 6:**
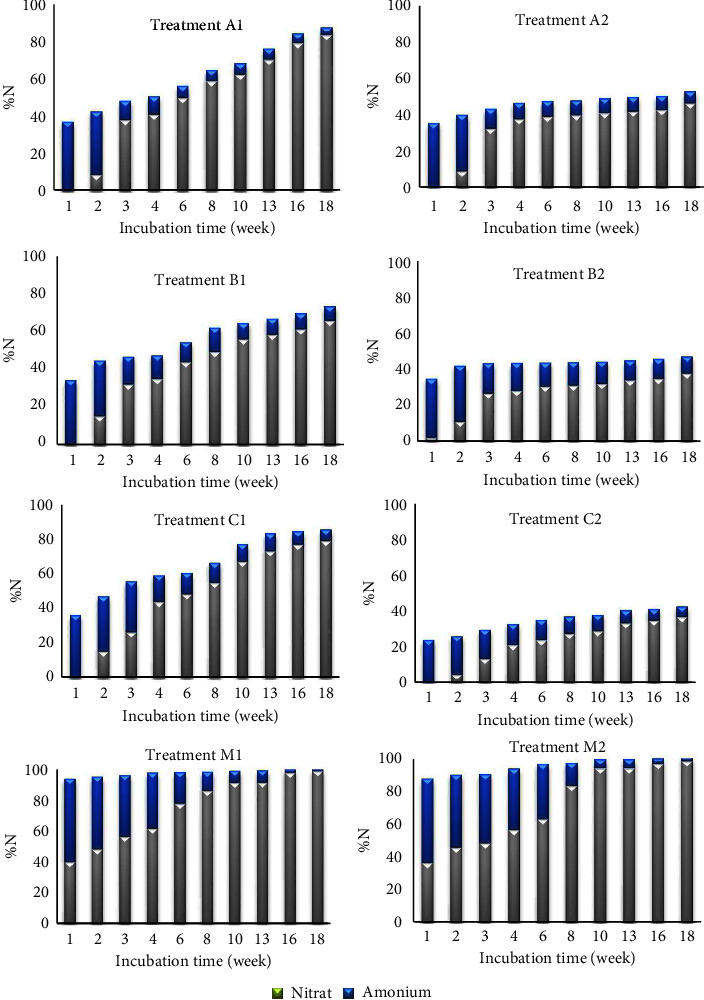
Diagram of nitrogen release (%) during 18 weeks of incubation.

The N release pattern in the eight fertilizer treatments in the form of a comparison between the percentage of NH_4_-N and NO_3_-N during the incubation time is presented in Figure 6.

#### 3.1.1. P Release Pattern

**Figure 7:**
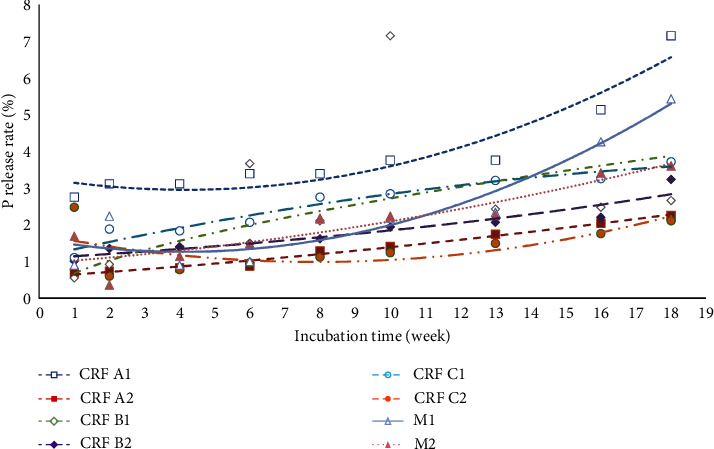
The release pattern of phosphorus (%) of the eight fertilizer treatments at 18 weeks of incubation.

P plays a role in increasing root development and as a source of energy by forming ATP [74]. P is absorbed by plants in the form of the H_2_PO_4_^–^ [75]. This study found that P release increased over time during the incubation period, but the amounts released varied. In the first week, P release from the fertilizers ranged from 0.55% to 2.75%, with CRF A1 releasing the most at 2.75%. By the 18th week, the release ranged from 0.55% to 7.16%, again with CRF A1 releasing the most at 7.16%. Importantly, there was a noticeable increase in release during the first two weeks of incubation across different treatments. This means that there was a clear rise in P release from Week 1 to Week 2, and these patterns are clearly illustrated in Figure 7, indicating an early increase in release during the incubation period.

While there was an increasing trend in P release until the 18th week of incubation, the release was not significant. This is demonstrated by the statistical analysis of the data in Table 6, where identical letters indicate no significant difference among all treatments. The release had an increasing trend until the 18th week of incubation, but the release of P was not significant. The released P will then be available in the soil and can be easily bound by other cations in the soil. P deficiency generally occurs in acid soils with high Fe and Al content due to fixation. However, treatment CRF A1 showed a significant difference compared to the other seven treatments, which should not have occurred. This disparity may be due to variability in the samples, with CRF A1 possibly having greater variability, affecting result consistency. Additionally, the smaller sample size for CRF A1 could contribute to increased variability in the results. Shi et al. [76] also showed that the heterogeneity of litter item characteristics varies between different litter categories, and that smaller sample sizes can lead to increased variability in the results.

The B1 treatment, incorporating CRF coating with a PEG dose of 0.31 g/100 g soil, demonstrated an optimized P release pattern up to the 8th week, followed by a stabilized release until the end of the incubation period. The results presented in Table 6 show a gradual increase in P release from the B1 treatment, starting at 0.55% in the first week and peaking at 7.16% in the 8th week, then stabilizing at 2.43%, 2.48%, and 2.66% in the 18th week. This gradual increase followed by stabilization indicates an optimized release pattern, maintaining consistent P availability with minimal variations.

In contrast, the CRF C2 treatment, utilizing CRF coating with a humic acid dose of 0.62 g/100 g soil, showed a notable initial release of P, followed by a decrease in the 2nd week, and then a constant increase until the 18th week of incubation. Table 6 shows that P release from the CRF C2 treatment started at 2.48% in the first week, decreased to 0.60% in the 2nd week, and then gradually increased, reaching 2.11% in the 18th week. This initial rapid rise followed by a consistent increase indicates a notable initial release of P, likely influenced by the specific characteristics of the humic acid coating.

Moreover, compared to N, P releases more slowly due to its immobility, with the coating on fertilizers having minimal impact on its release. In addition, the movement of P in the soil is very slow [77] due to the high reactivity of P with soil cations and P which is rapidly converted into organic P by soil microbial activity [18, 78, 79]. The released P will then be available in the soil and can be easily bind by other cations in the soil. P deficiency generally happens in acid soils that contain high Fe and Al content due to fixation [80].

### 3.2. K Release Pattern

K is a macro nutrient for plants that is needed in large quantities after N and P. K is a catalytic agent that plays a role in plant metabolic processes. The content and dynamics of soil K nutrients need to be known to determine the amount of fertilizer applied for efficient fertilization. According to [81], the efficiency ratio of K fertilizer addition must be calculated to estimate the amount of K retained in the soil.

**Figure 8:**
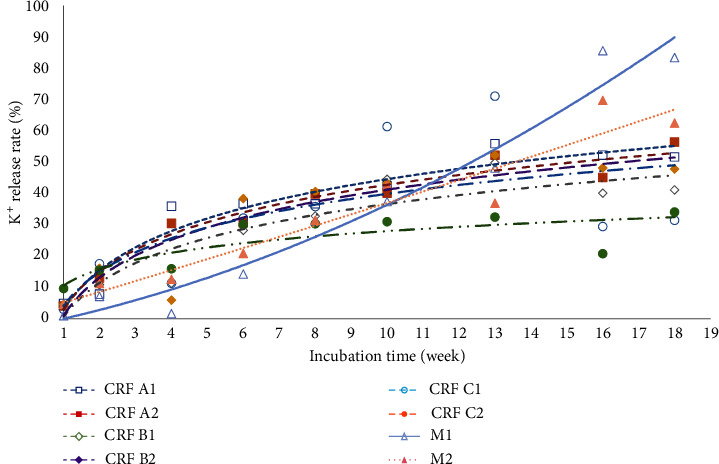
The release pattern of potassium (%) of the six CRF treatments at 18 weeks of incubation.

Figure 8 illustrates the K release pattern across the six CRF treatments over an 18-week incubation period. Overall, K release across the eight treatments followed a similar pattern. Statistical analysis indicated that the C2 treatment had the lowest mean K release (Table 7). This treatment released only about 24.48% of the total fertilizer K, likely due to the presence of humic acid. Humic acid has active functional groups of phenol, carboxylate, and aromatic which can decelerate the K release process compared to other treatments [82]. During the first week, K release from all treatments ranged from 3.04% to 9.73%. This release increased significantly to between 32.54% and 71.17% by the 13th week, before declining by the 18th week of incubation. The slower K release in the C2 treatment is attributed to the humic acid's functional groups, which provide negative charges and contribute to higher CEC and exchangeable K values [72].

Treatment M shows an increase in K release in the 18th week, particularly in treatments M1 and M2. M2, with a higher dose, reached 62.56%, while M1 reached 83.52%. This difference is due to the fact that M2 is not a CRF, resulting in faster and larger K release compared to other CRF treatments. Treatment M1, with a lower dose but achieving higher K release, also suggests that factors other than dose, such as fertilizer type, influence K release from the soil. Although the K content in treatment M is lower (16%) compared to CRF (27%), the K release in treatment M is higher due to factors such as nutrient release rate, coating type, physical and chemical properties of the fertilizer, soil interaction, and environmental conditions. CRF is designed to release nutrients slowly [83], so even though it has a higher K content, K release occurs more slowly compared to noncoated NPK Mutiara fertilizer.

Because of the absence of coating, CRF A1 and CRF A2 release K more quickly than coated CRF (CRF B1, CRF B2, CRF C1, and CRF C2). M1 and M2 release K quickly, especially in the later weeks, showing that NPK Mutiara 16:16:16 (M) releases K faster and in larger amounts than CRF. Despite its lower K content, the faster release rate of treatment M leads to a higher total K release in the later weeks of the study. The higher average K release observed in CRF A1 and CRF A2 (Table 7), despite their faster initial release, can be attributed to their higher K content and consistent release over the observation period.

### 3.3. Selection of Fertilizer Coating on CRF

Coating fertilizers with polymers is one of the most effective methods for achieving slow and controlled release into the soil [84]. This approach minimizes excessive fertilizer use and enhances nutrient availability to meet crop needs. Humic acid has been widely used as a reference in slow-release formulations of fertilizers. Previous research by [73] demonstrated that incorporating humic acid into slow-release fertilizers can significantly slow the release of N (as ammonium and nitrate) and K in the soil. In a recent study by Suwardi et al. [85], it was demonstrated that the release of N from NPK Granule Plus, which employs humic acid for slow release, generally decreases over the incubation period.

However, the efficacy of humic acid coatings can vary with dosage. When the dose of CRF was increased to 0.62 g/100 g soil, the N release rate decreased. However, the one-time dose (0.31 g/100 g soil) of humic acid coating was not efficient in releasing nutrients, highlighting the need to enhance the humic acid formulation. This improvement is essential to slow down the rate of N release among different fertilizer doses. Humic substances had the potential to improve osmolytes and nutrient homeostasis of plants under different growth conditions [86, 87]. Humic acid is commonly used as a component in slow-release fertilizers and is also widely employed in agriculture due to its proven ability to enhance the physical, chemical, and biological properties of soil [88–90].

In contrast, PEG is more commonly used in the pharmaceutical field and has not been widely developed in agriculture, so further tests are needed such as direct application to plants to see the direct reaction between fertilizers with plants [91]. Research by Khunkeaw et al. [92] has shown promising results with PEG coatings, significantly improving germination percentages and seedling vigor indices compared to uncoated seeds. Additionally, according to Pradana [93], coating PEG has proven to be the most effective method for slow-release fertilizer, resulting in significant increases in stem diameter and growth in oil palm. PEG enhances nutrient retention and slow release in fertilizers through its water solubility, high viscosity, and chemical stability. It binds nutrients to prevent rapid leaching, thickens fertilizer solutions to ensure prolonged availability, and resists degradation, maintaining effectiveness over time [64, 94].

Based on N release patterns observed in our study, CRFs with PEG coatings effectively delayed ammonification until the end of the incubation period. Comparatively, humic acid coatings (C1 and C2) exhibited slower nitrate release, particularly evident in C2 (CRF with humic acid at 0.62 g/100 g soil), which showed a 36.26% nitrate release and 24.48% K release by the end of incubation due to stable bonds formed by humic acid's functional groups. Overall, coated CRF demonstrates superior performance compared to noncoated fertilizers.

Treinyte et al. [95] explored biodegradable polymer composites made from polyvinyl alcohol (PVA), horn meal (HM), rapeseed cake (RC), glycerol (G), and phosphogypsum (PG), which can serve as nutrient-rich coating materials containing P, N, calcium, K, and sulfur. Factors influencing the selection of fertilizer coating on CRFs include the choice of coating material (e.g., permeability and biodegradability), environmental conditions (soil type, moisture, temperature, and pH), fertilizer formulation and dosage (nutrient composition and application rate), filler materials (e.g., HM, RC, and PG), and economic and environmental considerations (cost-effectiveness and sustainability). These factors collectively determine nutrient release rates, efficiency, and environmental impact, crucial for optimizing CRF performance in agricultural applications.

Understanding and optimizing these factors are essential for developing effective and economically viable solutions for slow-release fertilizers that meet both agricultural and environmental sustainability goals. Further research is needed to refine coating technologies and their application in diverse agricultural settings.

## 4. Conclusions

Coating CRF with PEG and humic acid impacts nutrient release compared to uncoated CRF and non-CRF treatments. Treatment M consistently showed the highest N, P, and K nutrient release due to its noncontrolled release nature. The B2 (CRF with PEG at 0.62 g/100 g soil) had a slower release of both ammonium and nitrate. Ammonium release started at 32.22% in the first week, decreasing to 9.36% by the 18th week, attributed to PEG's hygroscopic properties that absorb water first, preventing urea conversion to ammonium. Additionally, B2 initially had a lower rate of nitrate release, gradually increasing from 26.37% to 37.36% between the third and 18th weeks. However, C2 (CRF with humic acid at 0.62 g/100 g soil) demonstrated an even slower and lower release of nitrate, reaching 36.26% by the end of the incubation period, due to stable bonds formed by humic acid's functional groups. P release increased until the 18th week, with no significant difference between coated and uncoated CRF treatments, as the released P readily binds with other soil cations. Overall, K release patterns were similar across treatments, with C2 showing the lowest mean release at 24.48%, likely due to humic acid's influence. These findings highlight the potential of PEG and humic acid coatings to optimize nutrient release and improve fertilizer efficiency. Further investigation is needed to explore the long-term effects of these coatings on different soil types and crops.

## 5. Recommendations

To gain a comprehensive understanding of the effects of different coating materials on nutrient release, it is crucial to conduct additional biological testing to evaluate the dynamics of obligate autotrophic bacteria in the treated soil. This will help elucidate how these treatments influence soil microbial communities and nutrient cycling. Furthermore, future research should focus on applying CRF directly to plantation crops in real field conditions, such as oil palm, rubber trees, or similar plants. Investigating the performance of CRF in practical agricultural settings will provide valuable insights into its effectiveness and benefits, helping to optimize nutrient release and enhance crop productivity.

## Figures and Tables

**Table 1:** Types and dosages of fertilizers.

Treatment	Types of fertilizer	Dosages of fertilizer (g/100 g soil)
CRF A1	CRF 13-6-27 uncoated	0.31
CRF A2	CRF 13-6-27 uncoated	0.62
CRF B1	CRF 13-6-27 PEG coated	0.31
CRF B2	CRF 13-6-27 PEG coated	0.62
CRF C1	CRF 13-6-27 humic coated	0.31
CRF C2	CRF 13-6-27 humic coated	0.62
M1	NPK Mutiara	0.31
M2	NPK Mutiara	0.62

**Table 2:** Ammonium release (%) for different treatments over 18 weeks.

Treatment	Week										Means
	1	2	3	4	6	8	10	13	16	18	
CRFA1	37.01	33.82	10.21	9.57	6.38	5.96	5.96	5.74	5.10	3.83	12.36^c^
CRFA2	35.09	30.95	10.85	8.93	8.61	8.14	8.08	7.87	7.66	6.38	13.26^c^
CRFB1	34.46	29.35	14.68	12.34	10.63	12.76	8.61	8.29	8.29	7.66	14.71^bc^
CRFB2	32.22	30.52	16.59	15.31	13.08	12.76	12.12	10.85	10.85	9.36	16.37^abc^
CRFC1	36.37	31.69	29.35	15.31	12.34	11.49	10.21	10.21	7.66	6.38	17.10^abc^
CRFC2	33.82	30.31	22.97	16.27	15.95	13.40	12.76	10.21	8.93	8.29	17.29^abc^
M1	53.91	46.88	39.84	35.94	20.31	12.50	7.81	7.81	2.08	1.04	22.81^ab^
M2	51.56	44.53	42.19	37.50	33.59	14.06	5.47	5.47	3.13	2.34	23.98^a^

*Note:* The presented data are statistically significant. Differences with a significance level of *p* < 0.05 are denoted by different letters within the same column.

**Table 3:** Tukey simultaneous tests for differences of means.

Difference of fertilizer levels	Difference of means	SE of difference	Simultaneous 95% CI	*T*-value	Adjusted *p* value
CRFA2-CRFA1	0.90	2.75	(−7.73; 9.52)	0.33	1.000
CRFB1-CRFA1	2.35	2.75	(−6.28; 10.98)	0.85	0.989
CRFB2-CRFA1	4.01	2.75	(−4.62; 12.63)	1.46	0.827
CRFC1-CRFA1	4.74	2.75	(−3.88; 13.37)	1.72	0.673
CRFC2-CRFA1	4.93	2.75	(−3.69; 13.56)	1.79	0.628
M1-CRFA1	10.46	2.75	(1.83; 19.08)	3.80	0.008
M2-CRFA1	11.63	2.75	(3.00; 20.25)	4.22	0.002
CRFB1-CRFA2	1.45	2.75	(−7.17; 10.08)	0.53	0.999
CRFB2-CRFA2	3.11	2.75	(−5.52; 11.74)	1.13	0.948
CRFC1-CRFA2	3.84	2.75	(−4.78; 12.47)	1.40	0.856
CRFC2-CRFA2	4.04	2.75	(−4.59; 12.66)	1.47	0.822
M1-CRFA2	9.56	2.75	(0.93; 18.18)	3.47	0.020
M2-CRFA2	10.73	2.75	(2.10; 19.35)	3.90	0.006
CRFB2-CRFB1	1.66	2.75	(−6.97; 10.28)	0.60	0.999
CRFC1-CRFB1	2.39	2.75	(−6.23; 11.02)	0.87	0.988
CRFC2-CRFB1	2.58	2.75	(−6.04; 11.21)	0.94	0.981
M1-CRFB1	8.11	2.75	(−0.52; 16.73)	2.94	0.081
M2-CRFB1	9.28	2.75	(0.65; 17.90)	3.37	0.027
CRFC1-CRFB2	0.73	2.75	(−7.89; 9.36)	0.27	1.000
CRFC2-CRFB2	0.93	2.75	(−7.70; 9.55)	0.34	1.000
M1-CRFB2	6.45	2.75	(−2.18; 15.07)	2.34	0.289
M2-CRFB2	7.62	2.75	(−1.01; 16.24)	2.77	0.122
CRFC2-CRFC1	0.19	2.75	(−8.43; 8.82)	0.07	1.000
M1-CRFC1	5.71	2.75	(−2.91; 14.34)	2.07	0.442
M2-CRFC1	6.88	2.75	(−1.74; 15.51)	2.50	0.215
M1-CRFC2	5.52	2.75	(−3.10; 14.15)	2.00	0.487
M2-CRFC2	6.69	2.75	(−1.93; 15.32)	2.43	0.245
M2-M1	1.17	2.75	(−7.45; 9.80)	0.43	1.000

*Note:* Individual confidence level = 99.74%.

**Table 4:** Nitrate release (%) for different treatments over 18 weeks.

Treatment	Week										Means
	1	2	3	4	6	8	10	13	16	18	
CRFA1	0.00	8.79	38.10	41.03	49.82	58.61	62.27	70.33	79.12	83.52	49.16^b^
CRFA2	0.00	8.79	32.23	37.36	38.83	39.56	40.66	41.76	42.49	46.15	32.78^cd^
CRFB1	0.00	15.39	32.23	35.17	43.96	49.45	56.04	58.61	61.54	65.93	41.83^bc^
CRFB2	2.20	10.99	26.37	27.84	30.22	30.77	31.87	33.70	34.43	37.36	26.58^d^
CRFC1	0.00	15.39	26.37	43.96	48.35	54.95	67.03	73.26	76.92	79.12	48.53^b^
CRFC2	0.00	4.40	13.19	20.88	23.44	27.11	28.57	32.97	34.43	36.26	22.12^d^
M1	40.10	48.44	56.51	61.88	78.02	86.09	91.46	91.51	97.92	98.96	75.09^a^
M2	36.33	45.83	48.44	56.51	63.23	83.41	94.17	94.19	96.88	98.44	71.74^a^

*Note:* The presented data are statistically significant. Differences with a significance level of *p* < 0.05 are denoted by different letters within the same column.

**Table 5:** Nitrogen release (%) for different treatments over 18 weeks.

Treatment	Week										Means
	1	2	3	4	6	8	10	13	16	18	
CRFA1	37.01	42.61	48.30	50.60	56.20	64.56	68.23	76.07	84.23	87.34	61.51^b^
CRFA2	35.09	39.74	43.08	46.30	47.44	47.70	48.74	49.63	50.15	52.53	46.04^c^
CRFB1	34.46	44.74	46.91	47.50	54.59	62.21	64.66	66.90	69.83	73.59	56.54^b^
CRFB2	34.42	41.51	42.96	43.15	43.30	43.53	43.99	44.55	45.28	46.72	42.94^c^
CRFC1	36.37	47.08	55.72	59.27	60.69	66.43	77.24	83.47	84.58	85.50	65.64^b^
CRFC2	33.82	34.70	36.16	37.15	39.40	40.51	41.33	43.18	43.37	44.56	39.42^c^
M1	94.01	95.31	96.35	97.81	98.33	98.59	99.27	99.32	100.00	100.00	97.90^a^
M2	87.89	90.36	90.63	94.01	96.82	97.47	99.64	99.66	100.00	100.00	95.65^a^

*Note:* The presented data are statistically significant. Differences with a significance level of *p* < 0.05 are denoted by different letters within the same column.

**Table 6:** Phosphorus release (%) for different treatments over 18 weeks.

Treatment	Week									Means
	1	2	3	6	8	10	13	16	18	
CRFA1	2.75	3.12	3.12	3.39	3.39	3.76	3.76	5.14	7.16	3.95^a^
CRFA2	0.69	0.73	0.83	0.87	1.28	1.41	1.74	2.04	2.25	1.32^b^
CRFB1	0.55	0.92	1.38	3.67	2.11	7.16	2.43	2.48	2.66	2.59^b^
CRFB2	1.01	1.35	1.40	1.49	1.62	1.94	2.06	2.20	3.23	1.81^b^
CRFC1	1.10	1.88	1.83	2.06	2.75	2.84	3.21	3.26	3.72	2.52^b^
CRFC2	2.48	0.60	0.78	0.96	1.12	1.24	1.49	1.76	2.11	1.39^b^
M1	0.89	2.24	0.89	1.00	2.16	2.18	2.34	4.27	5.44	2.38^b^
M2	1.69	0.35	1.14	1.48	2.18	2.23	2.25	3.41	3.60	2.04^b^

*Note:* The presented data are statistically significant. Differences with a significance level of *p* < 0.05 are denoted by different letters within the same column.

**Table 7:** Potassium release (%) for different treatments over 18 weeks.

Treatment	Week									Means
	1	2	3	6	8	10	13	16	18	
CRFA1	4.87	7.91	35.99	36.80	36.80	42.58	55.96	52.31	51.76	36.11^a^
CRFA2	4.11	13.08	30.51	30.72	39.92	40.15	52.31	45.16	56.49	34.72^a^
CRFB1	4.56	10.95	11.35	28.28	32.85	44.61	49.88	40.15	41.21	29.32^a^
CRFB2	4.56	16.12	5.98	38.42	40.68	43.34	52.31	48.20	47.88	33.05^a^
CRFC1	3.04	17.64	11.35	32.24	35.89	61.43	71.17	29.50	31.47	32.64^a^
CRFC2	9.73	15.51	16.02	30.11	30.41	31.02	32.54	20.83	34.14	24.48^a^
M1	1.03	7.19	1.71	14.37	31.31	37.46	48.24	85.71	83.52	34.50^a^
M2	4.62	11.29	12.66	20.87	31.48	42.85	36.95	69.80	62.56	32.56^a^

*Note:* The presented data are statistically significant. Differences with a significance level of *p* < 0.05 are denoted by different letters within the same column.

## Data Availability

The data supporting this research article are derived from previously conducted studies and datasets, all of which have been appropriately cited within the article. The processed data can be accessed via the following repository: IPB Repository (https://repository.ipb.ac.id/handle/123456789/104333). Additionally, the supplementary files containing the data are available upon request from the corresponding author.
